# Effect of electron and X-ray irradiation on microbiological and chemical parameters of chilled turkey

**DOI:** 10.1038/s41598-021-04733-3

**Published:** 2022-01-14

**Authors:** Ulyana Bliznyuk, Valentina Avdyukhina, Polina Borshchegovskaya, Timofey Bolotnik, Victoria Ipatova, Zoya Nikitina, Alexander Nikitchenko, Igor Rodin, Felix Studenikin, Alexander Chernyaev, Dmitry Yurov

**Affiliations:** 1 Physics Department, Moscow State University, Moscow, 119991 Russia; 2 Skobeltsyn Institute of Nuclear Physics, Moscow State University, Moscow, 119991 Russia; 3 Chemistry Department, Moscow State University, Moscow, 119991 Russia; 4 National Scientific Research Institute of Medicinal and Aromatic Plants, Moscow, 117216 Russia; 5 Department of Epidemiology and Evidence-Based Medicine, I. M. Sechenov First Moscow State Medical University, Ministry of Health of the Russian Federation (Sechenov University), Moscow, 119991 Russia

**Keywords:** Biochemistry, Biophysical chemistry, Biomarkers, Predictive markers, Biochemistry, Biophysical chemistry, Reaction kinetics and dynamics, Nuclear physics, Experimental nuclear physics, Mass spectrometry

## Abstract

The purpose of this work was to compare the effect of electron and X-ray irradiation on microbiological content and volatile organic compounds in chilled turkey meat. Dose ranges which significantly suppress the pathogenic microflora while maintaining the organoleptic properties of the turkey meat are different for electron and X-ray irradiation. According to the study it is recommended to treat chilled turkey using X-ray irradiation with the dose ranging from 0.5 to 0.75 kGy, while in electron irradiation permissible doses should be within 0.25–1 kGy. Three main groups of volatile compounds: alcohols, ketones, and aldehydes—were found in irradiated and non-irradiated samples of turkey meat. It was found that the total amount of aldehydes, which are responsible for the formation of a specific odor of irradiated meat products, increases exponentially with the increase in the absorbed dose for both types of irradiation. It was established that acetone can be used as a potential marker of the fact of exposure of low-fat meat products to ionizing radiation.

## Introduction

Today, radiation technologies are increasingly used in the food industry in order to extend shelf life and ensure microbiological safety^1,2^. Choice of physical parameters, such as type of radiation, energy of charged particles, exposure time, intensity and density of particle fluence, particle current, one-side or two-side irradiation, plays an important role in food processing. A correctly selected irradiation scheme provides both the required absorbed dose and the best possible uniformity of depth dose distribution over the volume of the processed product.

For large volumes of dry products like tea, grain, spices, powders, it is advisable to use bremsstrahlung and gamma radiation sources with isotopes Co^60^ or Cs^137^, because somewhat uneven distribution of dose over the volume of processed products does not significantly affect the result of processing, as long as the selected irradiation technique is in compliance with recommended upper limit of the absorbed dose for these product categories^3^. While dry products do not require a strict control of dose uniformity, meat and fish products have to be treated within a rather narrow range to ensure the desirable dose uniformity throughout the entire volume. Since a more uniform distribution of the absorbed dose is required, electron radiation is the preferred treatment method when it comes to meat and fish provided that the irradiation method, operating mode of electron accelerator, and the thickness of the products are matched adequately^4^.

Under the influence of various types of radiation on organic products, various physical processes of interaction between radiation and matter occur, determining the nature of energy transfer to the product. Changes in physical and chemical parameters and subsequent organoleptic changes in food products, such as taste, color and smell can be different at the same doses for different types of radiation^5–7^. It is known that under the influence of ionizing particles, water molecules form free radicals (hydrogen atoms and hydroxyl radicals), that subsequently react with micro- and macromolecules of the product, which leads to the formation of new radicals and various chemical compounds. The difference in spatial distribution of free radicals under the influence of different types of irradiation leads to various effects on microorganisms inhabiting food products, as well as to the distribution of post-radiation biochemical processes of oxidation of fats and proteins in the product. It is known that the oxidation of fats leads to the formation of volatile compounds: alcohols, aldehydes, ketones and other oxygen-containing compounds^8–11^. It is interesting to study the composition of volatile compounds to compare the effect of various types of irradiation on microorganisms in the products exposed to the same dose range. As the result, it will be possible to find the optimal irradiation method for the given type of food products.

Different methods of chemical analysis are used depending on the type of product. For dry products, the electron-paramagnetic resonance technique is actively applied^12–15^. This method, however is not applicable to products containing a high amount of moisture, because it does not to detect free radicals after the treatment due to diffusion of radicals. It is possible to use “spin traps” to register radicals in moisture-containing products, but it is an expensive method that requires a lot of effort^16^. For products containing silicon, such as seafood, potatoes, onions, and beets, the methods of photostimulated luminescence and thermoluminescence are commonly used^17,18^. In foods with a high fat content, such as meat and seafood, the use of 2-thiobarbituric acid (2-TBA) tests and the analysis of hydrocarbons by gas chromatography have proved to be successful^19,20^.

These methods, along with other advanced methods of chemical analysis, represent an increasing interest for researchers who are seeking universal biomarkers that would make it possible to establish the fact of radiation treatment of various categories of food products^21–24^. The current international standard BS EN 1785–2003^20^ is based on the detection of 2-alkylcyclobutanones using gas chromatography that occur in meat products as a result of exposure to ionizing radiation. However, the detection of this chemical compound in low-fat meat products, such as turkey and chicken, is difficult due to the absence or a small content of this compound, therefore an alternative marker should be found to establish the occurrence of irradiation treatment^25^.

The purpose of this work is to compare the effect of electron and X-ray irradiation on microbiological properties and volatile organic compounds in chilled turkey meat treated with the same doses, as well as to search for a biomarker to identify the occurrence of irradiation treatment.

## Materials and methods

Food samples were irradiated by electron beam and X-rays at the same doses and dose rates. This study compared the effects of different types of radiation on volatile organic compounds and total microbiological parameters of food samples.

### Materials

Ten fresh, chilled turkey carcasses were bought from a local meat specializing in different kinds of poultry meat. Samples weighing 0.5 ± 0.1 g were taken from turkey breast and placed put in 2 ml individual plastic microcentrifuge tubes. For microbiological analysis 9 g of turkey meat were used from three different parts of each carcass. Similarly, 9 g of turkey meat from three different parts of each carcass were used for chemical analysis. In total, 180 g of turkey meat were used in both microbiological and chemical analyses. All manipulations were repeated for the experiment with electron and X-ray irradiation. Prior to the experiment, the samples had been stored in a refrigerator at a temperature of 2 °C for no more than 2 days post-slaughter.

### Experimental techniques

#### Electron beam treatment

Irradiation of chilled turkey samples was carried out at Skobeltsyn Institute of Nuclear Physics (Moscow, Russia) using continuous wave linear electron accelerator UELR-1-25-T-001 with an energy of 1 MeV and a maximum average beam power of 25 kW. This type of accelerator is used for industrial radiation processing of food and materials^26^. The beam current varied from 70 to 100 nA and the temperature of the radiation room was about 20 °C. The samples were irradiated from one side following the procedure described in^4^.

#### X-ray treatment

Two-side X-ray irradiation of chilled turkey samples was performed at Physics Department of Moscow State University using DRON UM-2 with power supply PUR5/50 and X-ray tube BSV-23 with a copper anode. The tube current was 26 mA and the voltage was 30 kV. The irradiation was carried out in the room with the ambient temperature of 20 °C following the procedure described in^4^.

### Dosimetry control

Ferrous Sulfate Solutions Dosimeter (Fricke dosimeter) was used for both electron and X-ray irradiation. The samples were irradiated with doses of 0.25 kGy, 0.5 kGy, 1 kGy, and 2 kGy. Dose rates of electron and X-ray irradiation were P_e_ = 1.2 ± 0.1 (Gy/s) and P_x_ = 5.6 ± 0.5 (Gy/s), respectively. As the obtained dose rates of electron and X-ray irradiation are close in value, it is possible to carry out a comparative assessment of the effect of different types of ionizing radiation in the same doses on the chemical and total microbiological parameters of chilled turkey.

### Dose uniformity assessment

The Geant4 program code based on Monte–Carlo method^27^ was used for the analysis of dose distribution in turkey samples after irradiation with accelerated electrons and X-rays.

### Electron beam dose uniformity ratio

**Figure 1:**
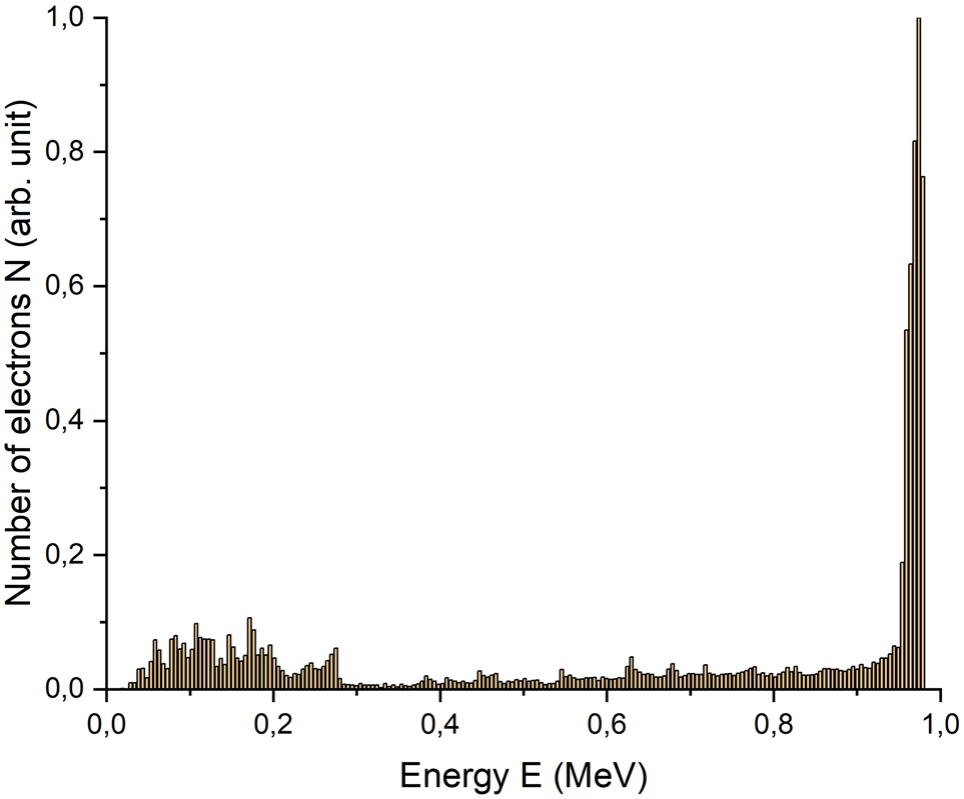
The electron spectrum N(E) of the accelerator UELR-1-25-T-001.

Simulations of the electron penetration into a water layer were performed by taking into account the electron spectrum of the UELR-1-25-E-001 (Fig. 1) as well as the irradiation method. The number of electrons in the beam was 10^9^. In cross-section, the bundle was a rectangle measuring 3 cm × 60 cm.

The 5 mm thick water phantom was divided into 50 layers 0.1 mm thick. The absorbed energy dE was recorded for each layer, and then the absorbed dose D in layers was determined using the following formula:

1 $${\mathrm{D}}_{\mathrm{l}ayer}=\frac{{\mathrm{dE}}_{layer}}{{\mathrm{dm}}_{layer}},$$

where dE_layer_ is the energy absorbed by the water layer, dm_layer_ is the mass of the layer.

**Figure 2:**
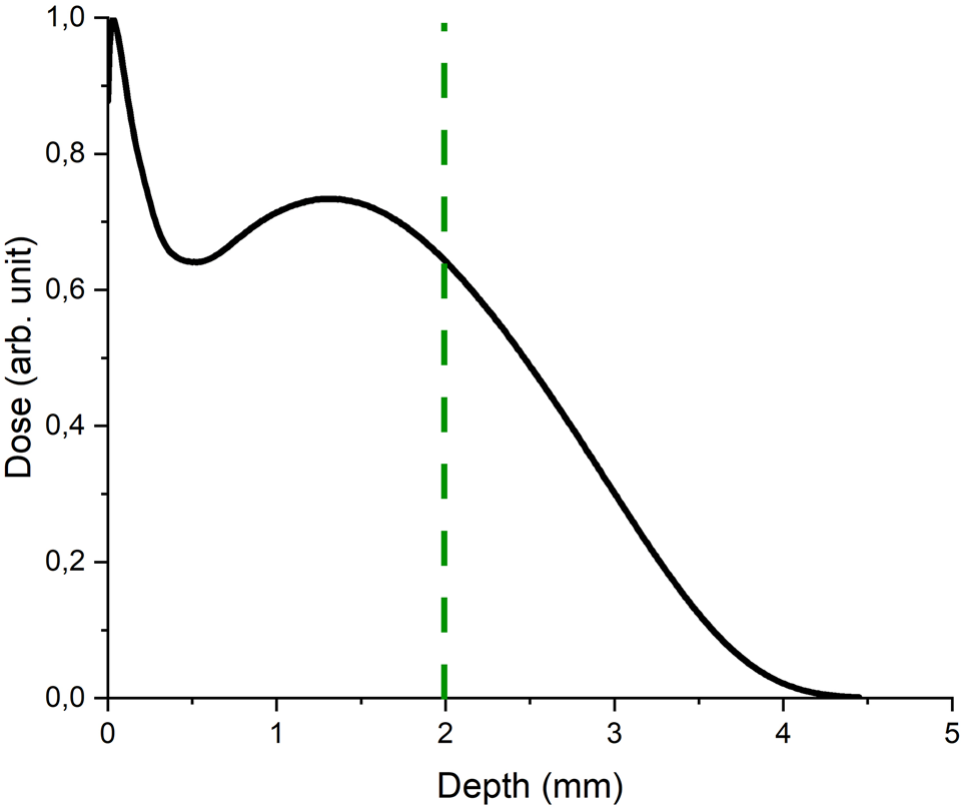
Depth dose distribution in a water layer 5 mm thick during 1 MeV electron treatment from one side. The dashed line is the average thickness of the turkey sample.

The dependence of the depth dose distribution in the water phantom was plotted based on the analysis of simulation results (Fig. 2).

Figure 2 shows that the depth dose distribution has an abnormal peak at the beginning of electron propagation in the water phantom. This peak at a depth of 0.1 mm is caused by the low-energy part of the electron accelerator spectrum (Fig. 1). Electrons with energies up to 100 keV, when interacting with molecules and atoms of a substance, lose approximately 0.5 keV per 1 μm of their path making a larger contribution to the dose at the initial part of the path than electrons of higher energies.

Uniformity of dose distribution, i.e. the ratio between the maximum and minimum dose along the direction of the electron beam equal to the maximum thickness of the sample, was about 65% and about 85% over the total volume of the 2 ± 0.5 mm thick samples.

### X-ray dose uniformity ratio

**Figure 3:**
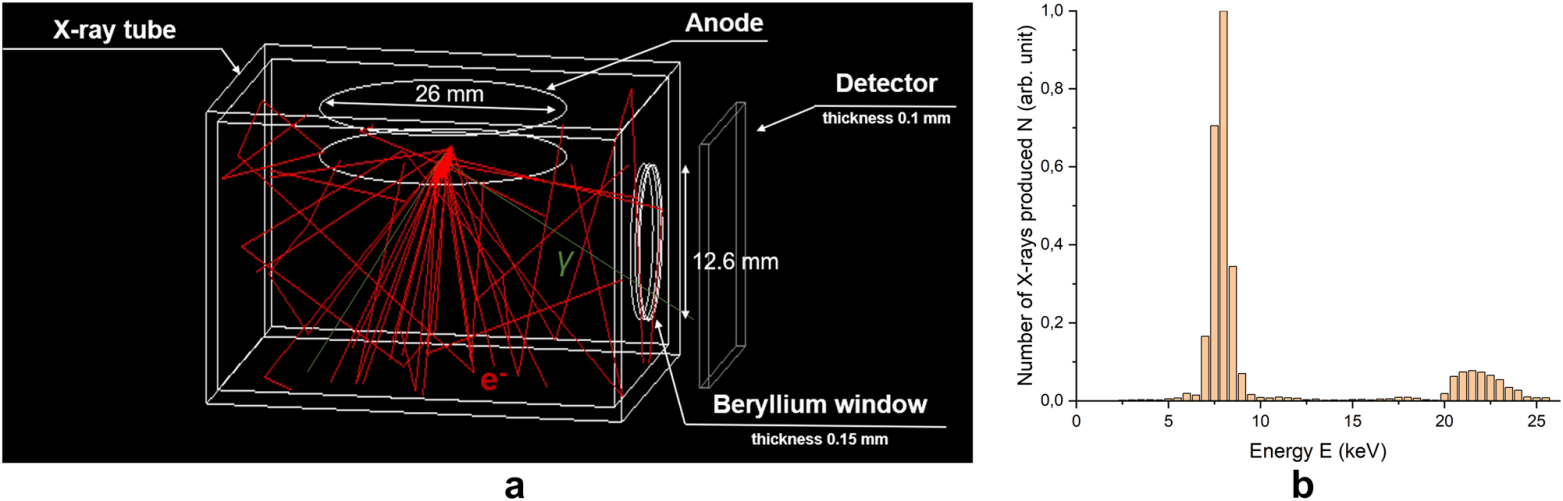
(**a**) Computer simulation of the BSV-23 Cu X-ray tube. Red lines are accelerated electrons and green lines are photons. (**b**) The spectrum of photons N(E) in arb. units at the output of the BSV-23 Cu X-ray tube.

The X-ray spectrum was calculated to estimate the dose distribution in the irradiated samples. During the simulation, all parameters of the BSV-23 Cu X-ray tube were reproduced with an accuracy of 0.1%. Figure 3a shows the computer simulation of the BSV-23 Cu X-ray tube, obtained using GEANT4 simulation toolkit version 4.10.5^27,28^.

In a vacuum lead chamber with walls 1 mm thick, a 26 keV electron beam was directed perpendicularly to a copper anode with a radius of 13 mm. The number of electrons in the beam was 10^9^. In cross-section, the bundle was a rectangle measuring 1 mm × 10 mm. Then the bremsstrahlung photons passed through a 12.6 mm ∅ and 0.15 mm thick beryllium window, and entered the water detector, which is a 16 mm × 16 mm and 0.1 mm thick parallelepiped, intended for registering the particles and their energies (Fig. 3b).

The photon spectrum has two peaks: the first one corresponds to characteristic radiation of copper, and the second one stands for the bremsstrahlung spectrum of X-ray irradiation (Fig. 3b).

Further, the transmission of two-side X-ray irradiation was simulated taking into account the calculated spectrum through a 7 mm thick water layer. The number of photons passing through the beryllium window was 10^9^. The turkey samples were simulated as 10 mm × 10 mm and 7 mm thick water parallelepipeds, located close to the beryllium window. The water phantom was divided into 70 layers with the thickness of 0.1 mm. The algorithm recorded the absorbed energy in each layer of water fantom and then recalculated it into the absorbed dose (1).

**Figure 4:**
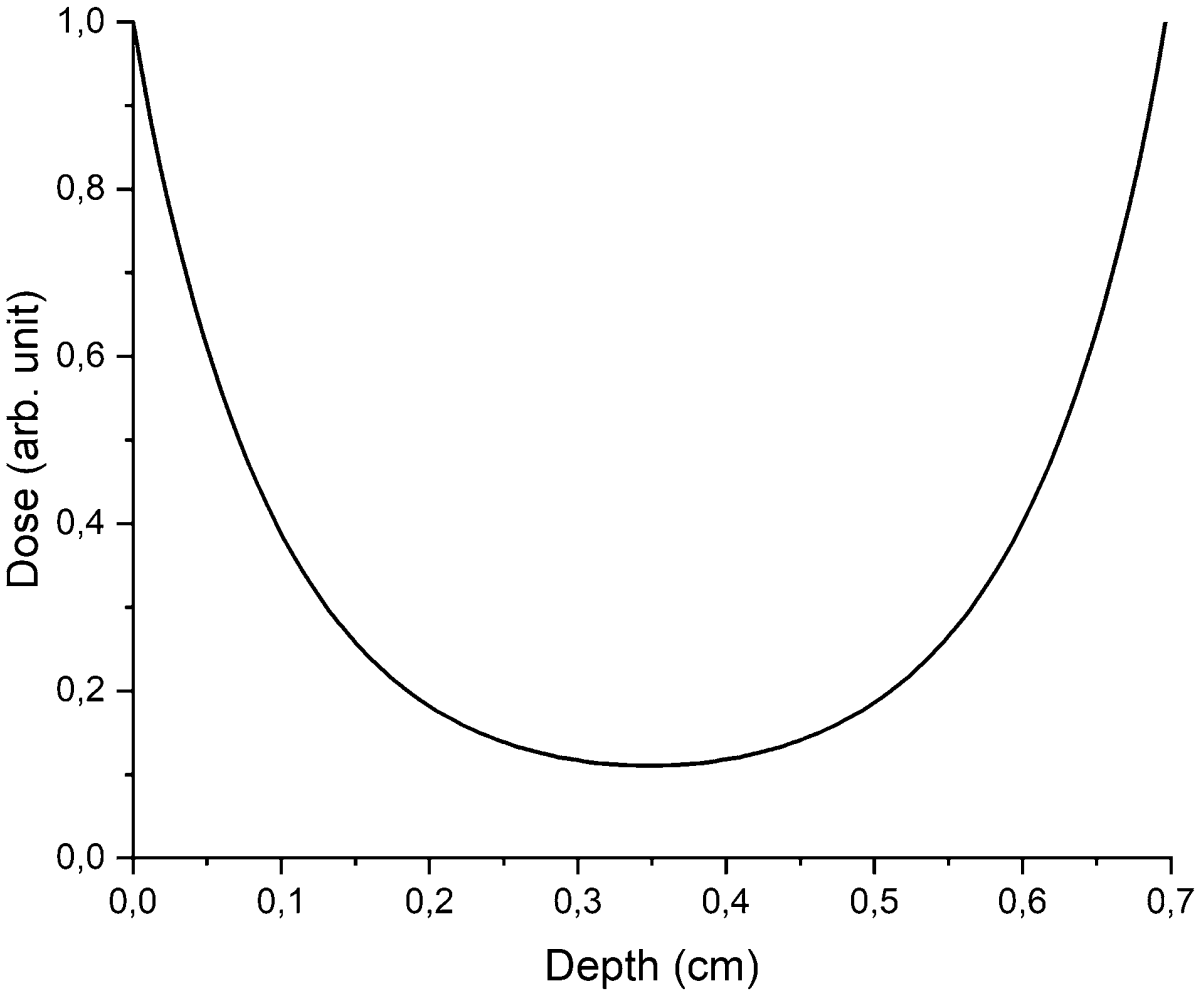
Depth dose distribution in a 7 mm thick water layer during two-side X-ray treatment.

Figure 4 shows the dependence of the depth dose distribution in the water phantom irradiated from two sides.

The uniformity of the dose distribution for a 7 mm thick water phantom was 15%. Since during the experiment turkey samples were placed in 7 mm ∅ cylindrical microcentrifuge tubes, the uniformity of the dose distribution over the total volume of the sample was significantly higher and amounted to 74%.

### Microbiological analysis

Total viable counts (TVC) of microorganisms in chilled turkey were determined according to^29^. The homogenate of turkey was diluted from 2 to 10,000 times in a physiological solution to obtain isolated cell colonies. After dilution, 0.1 mL of homogenate was spread-plated on Plate Count Agar. The concentration of TVC in the irradiated and non-irradiated samples was expressed as the number of colony-forming units per gram (CFU/g). All measurements and seeding were carried out under sterile conditions at 23 °C.

### Analysis of volatile compounds

Gas chromatography–mass spectrometry (GC–MS Shimadzu GCMS-QP2010 Ultra) was applied to analyze volatile compounds in irradiated turkey meat. Two grams of the meat sample and 4 mL of a sodium chloride (3% by weight) solution in distilled water were placed in a 20-mL vial and exposed to ultrasonic waves for 60 min. Next, the samples were subjected to thermal desorption at 95 °C for 20 min. To determine the various volatile compounds, 1 ml of the vapor phase of the sample was injected into the port of the GC–MS equipped with an automatic vapor phase injection device (HT200H Headspace Autosampler).

A capillary column (VF-624 MS, 60 m × 0.32 mm, 1.8 μm film thickness; J&W Scientific, USA) used to separate volatile compounds was initially programmed at 40 °C for 5 min; then the temperature was increased to 220 °C with the rate of 6 °C/min for 5 min. Helium, used as a carrier gas, had a flow rate of 1.5 cm^3^/min. The temperature of the evaporator, interface, quadrupole, and ion-source was maintained at 250, 200, 200, and 230 °C, respectively. Electron ionization mass spectra were taken at 70 eV. Chromatograms were recorded in the all-ion scan mode for m/z values from 33 to 350 with 3.3 scans/s. Identification of detected compounds was performed by comparing the obtained mass spectra with those from the NIST/EPA/NIH Mass Spectral Library 2008 (NIST 08)^30^.

### Statistical analysis

The data obtained were analyzed using OriginPro Learning Edition (OriginLab Corp., Northampton, Massachusetts). Each sample was evaluated in triplicates.

## Results and discussions

**Figure 5:**
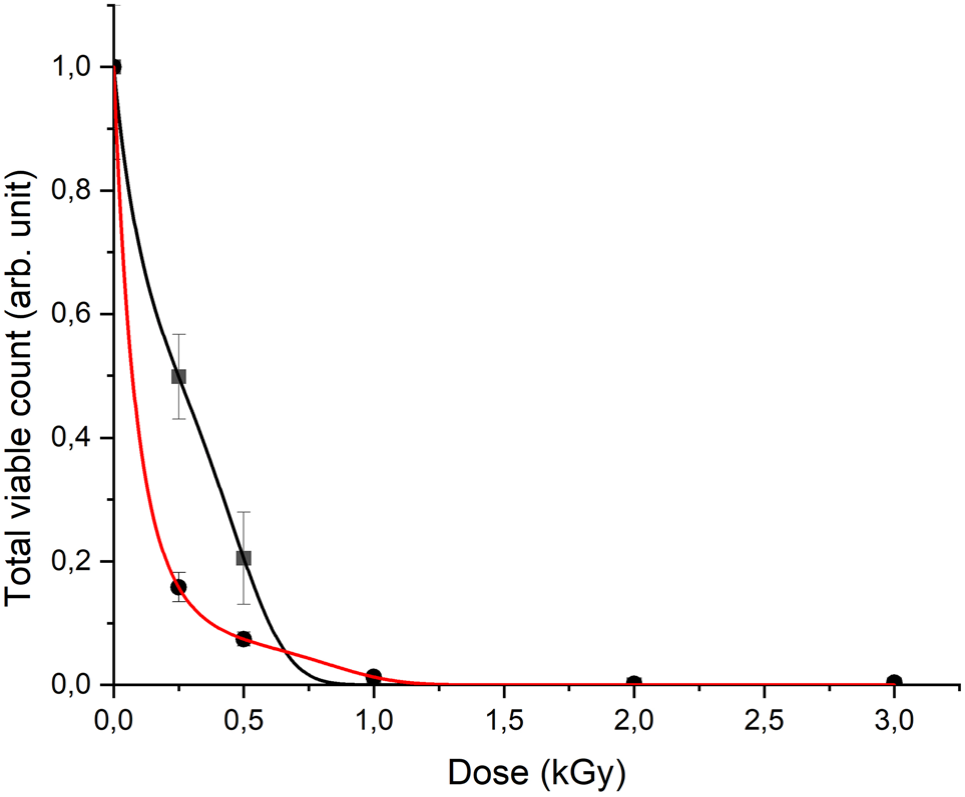
Dependences of the relative total viable count of microorganisms in chilled turkey meat on the dose of X-ray (black curve) and electron (red curve) radiation.

Concentration of microorganisms in turkey meat exponentially decreased with the increase in the dose of electron and X-ray radiation (Fig. 5). The initial concentration of microorganisms in the chilled Turkey at the start of our investigation was 10^3^ CFU/g, which is ten times lower than the upper limit of microbial contamination allowed for this category of food products^31^.

The dependence of total viable count of microorganisms from radiation dose is best described by the function:

2 $$f\left(D\right)={e}^{a+bD+c{D}^{2}+d{D}^{3}+o({D}^{3})},$$

where *D* is an absorbed dose of radiation, *a* is the determines the vertical position of the curve, *b*, *c*, and *d* reflects the rate at which the number of viable cells in the turkey decreases with the increase in the absorbed dose. The values of function f (D) were calculated with the least squares method: *a*_*e*_ = (4.01 ± 0.01) × 10^–7^ arb.un., *b*_*e*_ = − 10.7 ± 0.3 Gy^–1^, *c*_*e*_ = 15.7 ± 1.1 Gy^–2^, *d*_*e*_ = − 9.3 ± 0.9 Gy^–3^ for electron irradiation and *a*_*x*_ = (− 5.02 ± 0.01) × 10^–8^ arb.un., *b*_*x*_ = − 4.5 ± 0.2 Gy^–1^, *c*_*x*_ = 10.1 ± 1.4 Gy^–2^, *d*_*x*_ = − 15.6 ± 1.9 Gy^–3^ for X-ray irradiation*.* The correlation coefficients *R*_*e*_ and *R*_*x*_ were 0.999, which indicates the adequacy of the proposed approximation.

Figure 5 shows that for doses up to 0.5 kGy accelerated electrons with the dose rate 1.2 ± 0.1 (Gy/s) lead to a greater decrease in the concentration of TVC in turkey meat compared to X-rays. The reduction in the concentration of microorganisms to the 10^2^ CFU/g allows to extend the shelf life of poultry to 8 days compared to 4 days for non-irradiated samples^32^. This concentration was observed in turkey samples treated with electron radiation at a dose of 0.5 kGy. It is also can be seen in Fig. 5 that for X-ray irradiation this level of reduction in microorganisms is achieved at the dose of 0.75 kGy and above. At the dose of 1 kGy, the microbiological parameters in both cases decreased by more than 100 times compared to the control samples. Starting with a dose of 2 kGy, no viable microorganisms were found in turkey samples for either type of radiation.

Chemical investigation of turkey samples with gas chromato-mass spectrometry revealed three main groups of volatile compounds: aldehydes, ketones, and alcohols. Table 1 displays all identified volatile organic compounds in the studied samples as well as their amount in mg/kg irradiated with X-rays and electrons for various doses.

**Table 1:** The content of identified volatile compounds in the turkey samples (mg/kg; n = 3 is the number of repeats, P = 0.95 is the confidence level).

Class	Name		Control	0.25 kGy	0.5 kGy	1 kGy	2 kGy
Aldehydes	Pentanal	Electron	0.028 ± 0.007	0.042 ± 0.008	0.04 ± 0.01	0.05 ± 0.01	0.06 ± 0.01
		X-ray		0.023 ± 0.006	0.036 ± 0.009	0.04 ± 0.01	0.07 ± 0.02
	Hexanal	Electron	0.21 ± 0.05	0.4 ± 0.1	0.4 ± 0.1	0.4 ± 0.1	0.5 ± 0.1
		X-ray		0.18 ± 0.04	0.37 ± 0.09	0.36 ± 0.09	0.7 ± 0.2
Ketones	Acetone	Electron	ND ( ND – Not Detected.)	0.04 ± 0.01	0.04 ± 0.01	0.05 ± 0.01	0.07 ± 0.02
		X-ray		0.04 ± 0.01	0.04 ± 0.01	0.041 ± 0.009	0.05 ± 0.01
	2,3-Butanedione	Electron	1.5 ± 0.4	1.4 ± 0.4	0.5 ± 0.1	0.4 ± 0.1	0.6 ± 0.1
		X-ray		0.20 ± 0.05	0.21 ± 0.05	0.26 ± 0.07	0.30 ± 0.07
	3-Hydroxy-2-butanone	Electron	0.4 ± 0.1	0.18 ± 0.04	0.19 ± 0.05	0.10 ± 0.02	0.07 ± 0.02
		X-ray		0.28 ± 0.07	0.31 ± 0.08	0.6 ± 0.1	0.6 ± 0.2
	6-Methylheptanone-3	Electron	0.07 ± 0.02	0.04 ± 0.01	0.05 ± 0.01	0.06 ± 0.01	0.07 ± 0.02
		X-ray		0.08 ± 0.02	0.09 ± 0.02	0.12 ± 0.03	0.21 ± 0.05
Alcohols	Isopropanol	Electron	ND	0.037 ± 0.009	0.05 ± 0.01	0.053 ± 0.013	0.05 ± 0.01
		X-ray		0.08 ± 0.02	0.07 ± 0.02	0.08 ± 0.02	0.09 ± 0.02
	2,3-Butanediol	Electron	0.02 ± 0.004	0.020 ± 0.005	ND	ND	ND
		X-ray		0.22 ± 0.06	0.33 ± 0.08	0.4 ± 0.1	0.5 ± 0.1
	Hexanol-1	Electron	0.07 ± 0.02	0.06 ± 0.02	0.034 ± 0.008	0.04 ± 0.01	0.05 ± 0.01
		X-ray		0.06 ± 0.02	0.12 ± 0.03	0.14 ± 0.03	0.31 ± 0.07
	2-Ethylhexanol-1	Electron	0.08 ± 0.02	0.06 ± 0.01	0.05 ± 0.01	0.054 ± 0.013	0.11 ± 0.03
		X-ray		0.08 ± 0.03	0.09 ± 0.04	0.12 ± 0.03	0.21 ± 0.03

*ND* not detected.

Chilled turkey meat contains water, fats, proteins, amino acids, and vitamins, among other compounds. When the water fraction is exposed to ionizing radiation, free radicals are formed in it, which, in the presence of oxygen, initiate various oxidation reactions of organic molecules. Lipids, which contain saturated and unsaturated fatty acids, whose share in meat products is quite large, are the main target of oxidative processes for doses up to 1–2 kGy^33–36^. Fatty acids break down into alcohols, which, in turn, break down into aldehydes and ketones.

For each volatile compound, processes of accumulation, as a result of the disintegration of other organic compounds, and decay into other compounds are observed. These processes also occur when the product is in storage, and their intensity is a function of the temperature, concentration of oxygen, water, etc. After exposure to ionizing radiation, the intensity of the reactions of accumulation and decay of volatile compounds increases.

Various dependencies of alcohols (which are products of fatty acid decay) from the absorbed dose were identified for electron and X-ray irradiation. A similar dependence of the concentration from the dose of radiation in both types of radiation was identified for isopropanol, and it can be described as a function *f(D)*:

3 $$f\left(D\right)=a+b\left(1-{e}^{-cD}\right),$$

where *a* is the initial concentration of the compound, *b* shows the difference between the initial concentration of the studied compound and the final concentration, and *c* is saturation rate of the compound. The parameters were *a*_x_ = (1.16 ± 0.05) × 10^–7^ arb.un., *b*_x_ = 0.9 ± 0.6 arb.un., *c*_x_ = 30.2 ± 9.5 Gy^–1^ for X-ray irradiation, *a*_e_ = 0.003 ± 0.001 arb.un., *b*_e_ = 0.99 ± 0.06 arb.un., *c*_e_ = 5.1 ± 0.9 Gy^–1^ for electron irradiation. Correlation coefficients were 0.98 and 0.99, respectively. Isopropanol (Table 1) was not detected in the control samples or its concentration was negligible compared to the irradiated samples. At the same time, its concentration was virtually unchanged in the given dose range. The total amount of volatile compounds in chilled turkey meat after electron irradiation was higher than after X-ray irradiation. However, the concentration of some compounds, such as isopropanol, was higher for X-ray irradiation compared to electron irradiation.

While the dependence of concentration of alcohols 2-ethylhexanol-1 and isopropanol is similar in the given dose range for both types of irradiation, hexanol-1 is dramatically different when it comes to electron and X-ray irradiation.

Aldehydes are products of oxidation of alcohols. They are responsible for the formation of off-flavors and odors in the meat products. The dependence of total concentration of aldehydes on the dose for both electron and X-ray radiation can be described by the function (3). The parameters are *a*_x_ = 0.97 ± 0.01 arb.un, *b*_x_ = 65.3 ± 2.9 arb.un, *c*_x_ = 0.019 ± 0.003 Gy^–1^ for X-ray irradiation, and *a*_e_ = 1.1 ± 0.2 arb.un., *b*_e_ = 0.87 ± 0.09 arb.un., *c*_e_ = 1.86 ± 0.19 Gy^–1^ for electron irradiation. Values of correlation coefficients are *R*_*x*_ = 0.98, *R*_e_ = 0.95, respectively.

**Figure 6:**
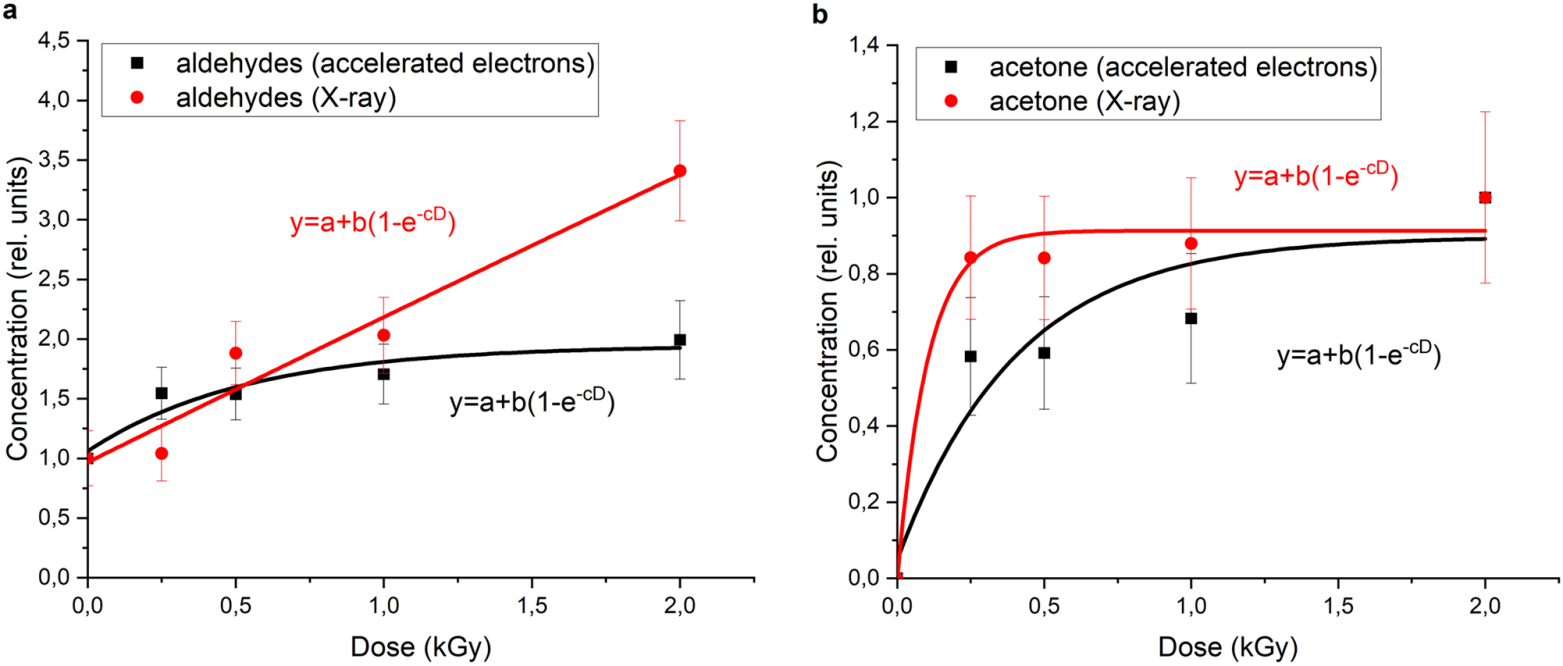
Dependence of the concentration of (**a**) aldehydes and (**b**) acetone on the dose of electron and X-ray radiation.

At the same time, the increase in the rate of the total concentration of aldehydes is higher for X-ray irradiation than for accelerated electrons (Fig. 6a). The high rate of aldehyde accumulation for X-ray irradiation can be explained by an intensive conversion of alcohols for doses ranging from 0.25 to 2 kGy. In contrast, after electron irradiation the process of aldehyde accumulation is compensated by their disintegration into other chemical compounds.

Ketones, which are also products of alcohol oxidation, display various dependencies on the doses in both types of irradiation (Table 1). For example, the concentration of 3-hydroxy-2-butanone, which is an intermediate product of oxidation of 2.3-butanediol to 2.3-butanedione, decreases with an increase in the dose of electron radiation. This dependency can be described as function *f(D)*:

4 $$f\left(D\right)=a+b{e}^{-dD},$$

where *a* is the initial concentration of 3-hydroxybutanone-2, *b* shows the difference between the initial concentration of 3-hydroxybutanone-2 and its final concentration, and *d* is the rate of decomposition of 3-hydroxybutanone-2. The parameters and correlation value are as follows: *a*_e_ = 0.19 ± 0.08 arb.un., *b*_e_ = 0.8 ± 0.1 arb.un., *d*_e_ = 1.2 ± 0.8 Gy^–1^, *R*_e_ = 0.97.

In the case of X-ray, there was a slight decline at dose 0.25 kGy and after that some growth was observed at a higher dose (Table 1). It indicates the dominance of decay of the compound at lower doses, an increase of accumulation rate at higher doses due to the breakdown of alcohols, and, finally, two competing processes reaching equilibrium.

Concentrations of other ketones, such as 2.3-butanedione and 6-methylheptaneone-3, also display various patterns for two types of radiation. However, the concentration of acetone, a product of isopropanol oxidation, increased with an increase in the dose for both types of radiation. This dependence can be described by (3) where *a*_x_ = 0.0010 ± 0.0003 arb.un., *b*_*x*_ = 0.91 ± 0.08 arb.un., *c*_x_ = 9.6 ± 0.2 Gy^–1^ for X-ray radiation, *a*_e_ = 0.05 ± 0.01 arb.un., *b*_e_ = 0.8 ± 0.2 arb.un., *c*_*e*_ = 2.5 ± 0.9 Gy^–1^ for electron radiation. Correlation coefficients were 0.99 and 0.94, respectively. At the same time, a high rate of the accumulation of compound was observed for electron radiation (Fig. 6b). Acetone was not detected in the non-irradiated turkey meat samples or its concentration was less than the error of the method for determining the concentration of volatile compounds. This suggests that it is possible to use this ketone as a marker to identify low-fat meat products that underwent radiation processing.

Different patterns of volatile compound concentration in different types of radiation can be explained by many factors. Volatile compounds, which are the product of the decay of fatty acids, are a result of the rupture of chemical bonds due to energy transfer to molecules of the substance. The nature of the interaction between electrons with the energy of 1 MeV and photons with a maximum energy of 26 keV with biological structures is different. For electrons, ionization predominates. At the beginning of the track of electrons with an energy of 1 MeV they lose more than half of their kinetic energy in frontal collisions with atoms of matter, accompanied by the knocking out of low-energy electrons with energy of about 1 keV, forming spurs in their wake—compact groups of pairs of ions (from 1 to 5), located inside some finite, probably spherical volume. Since low-energy electrons are capable of ionization, they form the so-called “branches of ionization” in directions other than the original direction of primary electrons. As the primary electrons pass through the substance, they lose the remaining half of their energy in collisions with molecules, with the average energy of loss being 30–100 eV. The spurs created by these electrons get closer and closer to each other, and eventually begin to overlap, forming cylindrical columns with a high density of ionization. At the end of the track of the primary electron, the movement of electrons becomes thermal and diffusive and produces a large number of acts of ionization. At the same time, as the energy of the primary electron decreases, ionization losses per unit of its path go up.

When X-rays with energies up to 26 keV interact with the product, electrons of various energies up to 26 keV are formed as a result of the photo effect and the Compton effect. Their acts of ionization are concentrated mainly in isolated spurs and branches of ionization. As a result of the heavy scattering, these acts of ionization are evenly distributed in the sample volume.

Because of this, the different nature of the interaction between electrons and X-rays with the substance leads to a different distribution of energy in the volume of irradiated samples and different distribution of chemical effects. However, there is a large number of general patterns for both types of radiation, such as an increased concentration of aldehydes and accumulation of acetone.

**Figure 7:**
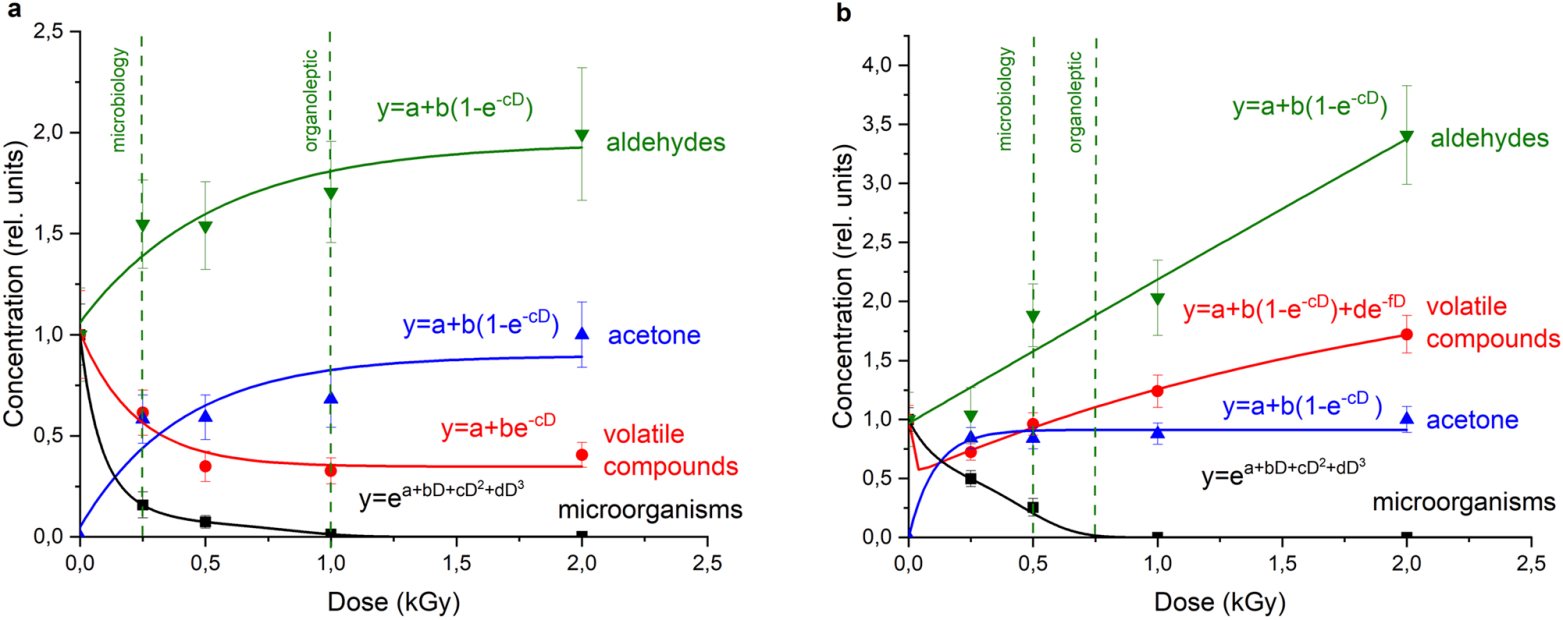
Dose dependence of the change in the relative concentration of volatile compounds (red curve), acetone (blue curve), aldehydes (green curve) and microorganisms (black curve) in turkey meat after treatment with (**a**) electron and (**b**) X-rays.

The dependence of relative concentrations of common volatile compounds, acetone, aldehydes, and TVC of microorganisms in the turkey from the dose of radiation for electronic and X-rays is displayed in Fig. 7.

The analysis of the dependence of the total concentration of aldehydes (Fig. 7a) reveals that the concentration of aldehydes increases 1.75–2 times in turkey samples processed by accelerated electrons for doses up to 1 kGy. From our previous organoleptic investigation, detailed in^4^, it can be seen that the exposure to accelerated electrons for doses up to 1–2 kGy has no noticeable effect on the appearance, taste, color or smell of turkey meat. The analysis of microbiological, organoleptic, and chemical parameters suggests that the recommended dose range for chilled turkey meat varies from 0.25 to 1 kGy for 1 MeV accelerated electron irradiation with dose rate 1.2 ± 0.1 (Gy/s).

The influence of X-rays, on the other hand, displays a more intense growth rate of aldehyde concentration to compare with electron irradiation. For X-ray radiation with a maximum photon energy of 26 keV and the power of the X-ray tube 1.8 ± 0.2 (Gy/s), the recommended range is from 0.5 to 0.75 kGy.

## Conclusion

Our research established the dose ranges of electron beam and X-ray irradiation which efficiently decrease the bacterial content in chilled turkey meat without significantly impacting the total concentration of volatile chemical compounds.

It is recommended that in the case of X-ray irradiation with the dose rate of 1.8 ± 0.2 (Gy/s) chilled turkey is treated with the dose ranging from 0.5 to 0.75 kGy, while in electron irradiation with the dose rate of 1.2 ± 0.1 (Gy/s), permissible doses should be within 0.25–1 kGy. The recommended ranges for electron and X-ray irradiation are established taking into account that the lowest dose levels for each type of irradiation sufficiently decrease microorganism content while the highest dose levels do not have an impact on the appearance, taste, color or smell of turkey meat, which was proved by organoleptic and microbiological analyses.

It was found that alcohols and ketones, which are products of lipid oxidation, display various dependencies on the doses for both types of irradiation. It was determined that after the X-ray or electron irradiation treatment, the concentration of acetone in irradiated samples goes up with an increase in the dose, despite the fact that acetone is not detected in the control non-irradiated samples. This makes it possible to use acetone as a potential marker of the fact of exposure of low-fat meat products to ionizing radiation.
